# Global Health Initiatives and Universal Health Coverage in Pakistan‐Aligned for the Future?

**DOI:** 10.1002/hpm.70038

**Published:** 2025-11-19

**Authors:** Shehla Zaidi, Shifa Salman Habib, Asad Shoaib, Zakir Shah, Karl Blanchet, Rosemary Jouhad, Natasha Palmer, Valery Ridde, Sophie Witter

**Affiliations:** ^1^ Aga Khan University Karachi Pakistan; ^2^ University College London London UK; ^3^ University of Geneva Geneva Switzerland; ^4^ Queen Margaret University Edinburgh UK; ^5^ Institut de Recherche pour le Développement Marseille France; ^6^ Cheikh Anta Diop University Dakar Senegal

**Keywords:** gavi, GFATM, GFF, global health initiatives, Pakistan, primary health care, universal health coverage

## Abstract

There is increasing global discourse on Global Health Initiatives' (GHIs) role and the need for better alignment with universal health coverage (UHC), which is particularly salient given recent rapid reductions in global aid. However, tensions within national ecosystems of GHI assistance and country alignment towards UHC are less well understood. We identify challenges and leverage points for aligning GHIs' assistance towards UHC‐focused health systems in Pakistan, drawing from the perspective of country stakeholders. A political economy approach was applied to unpack the context of national aid architecture, country discourse on strengths and weakness of GHI country ecosystem and stakeholders' power, positioning and interests for future reforms. Key informant interviews were conducted with constituencies of country‐based stakeholders in federal and provincial health systems, supplemented by a desk review of health financing data and policy‐programmatic documents.

The findings highlight a context of expanding GHI mandate, despite Pakistan's trajectory towards middle income country status, but weak alignment with national primary health care (PHC) budgeting and planning processes. Country discourse acknowledged improved disease coverage but surfaced tensions with the off‐budget parallel grant model, comprising of several GHI intermediaries, headquarters‐driven planning and selective system support, that was not positioned to build sustainability resulting in duplicative resourcing, questionable value for money, clouding of accountability roles and poor preparedness for transition. Competing interests between federal and provincial governments, and between disease managers and PHC planners, was perceived to further weaken country stewardship of GHI assistance. The prospect of an impending decline in aid funding was a common interest for change across all stakeholder constituencies. Stakeholders were positioned for a continuation of GHI assistance but with fundamental changes involving integration with national PHC budgeting, re‐balancing power through shared accountability, and calibrated federal‐provincial incentives for coordinated working, but most felt disempowered to bring about change.

We conclude that addressing power imbalances must be at the centre of paradigm shifts in country assistance by GHIs, although contextual modalities will differ across LMICs. Direct engagement with UHC stakeholders under the ambit of national PHC reforms, fewer intermediaries, on‐budget incentives to sustainably grow domestic financing and PFM technical assistance for aid management emerged as key areas for efficient, sustainable alignment in Pakistan and similar LMICs. Urgent actions are required within the current context of changes in global aid to build local capability for systematically easing dependence on GHIs while protecting equitable gains in disease outcomes.

## Highlights

- The political economy perspective is useful in providing deep dive insights within LMIC contexts to explore margins of change to better position GHI assistance within national PHC planning and budgeting.
- GHI assistance model of parallel funding exacerbates power imbalances between GHIs and country governments, erodes country leadership capacity, promotes fragmentation and heightens inefficiencies within scare resources, however the prospect of future decline in development assistance is a powerful lever to mobilise stakeholders for sustainable financing with more local accountability.
- The existing ecosystem of centralised parallel grant‐based aid disbursement models creates tensions and role confusion between federal and sub‐national governments, as well as between PHC planners and disease managers.
- GHIs assistance must be urgently re‐positioned to introduce calibrated incentives for growing domestic disease control funding, a shift to on‐budget support to national PHC budgets, investment in local coordination to support country driven solutions and widening technical assistance from disease metrics to public finance management for disease financing in LMICs.
- With rapid change in global aid order, the pace of change must be speeded up to enable local leadership in LMICs to efficiently protect gains, avoiding abrupt disruptions.

## 1 Introduction

There has been a significant increase in development assistance for health (DAH) disbursed through Global Health Initiatives (GHIs) to low‐middle income countries (LMICs) but efficiency, value for money and sustainability are increasingly being questioned [1]. GHIs are international partnerships that were put in place in the early 2000s to accelerate the response to priority communicable diseases as well as maternal and child health by mobilising and channelling disease‐oriented health financing [2]. One of the main concerns related to GHI assistance is the selective support to health systems linked to the delivery of specific targeted technical solutions [3, 4]. Another concern relates to whether the process by which GHIs provide country assistance is conducive in preparing LMICs for transition from external assistance to domestic financing [5]. These concerns are fundamentally linked to the verticalized grant based country funding model adopted by several GHIs that runs in parallel to national planning and financing cycles and contributes to the fragmentation of financing for impactful, efficient, sustainable primary health care within universal health care (UHC) [6, 7]. Recent online multi‐country surveys conducted by WHO underscore that country governments want better aid planning [8], but there is little in‐depth country research on how GHI assistance can be better aligned with the recent push for country‐led UHC‐oriented health systems.

Pakistan is a low‐middle income country that ranks amongst the main beneficiaries of GHIs' channelled assistance for disease control. The country is of strategic importance for the Vaccine Alliance (Gavi) and the Global Fund to Fight Tuberculosis, Malaria, HIV (GFATM) having the fifth largest burden of tuberculosis (TB), high levels of malaria, focal outbreaks of HIV infection with a high risk of generalised spread and incomplete routine childhood immunisation [9, 10, 11, 12, 13]. The Global Financing Facility for Women, Children, and Adolescents (GFF) is a more recent entrant in Pakistan. Overall donor support is less than 2% of overall total health expenditure but Gavi and GFATM support is not well documented and important for supporting priority communicable diseases that face historically low country allocations [14]. Despite substantial investment by GHIs for priority issues, country outcomes and country allocation remain below expectations, raising questions of how impact and sustainability can be maximised. On a parallel footing, UHC efforts have gained momentum in Pakistan supported by domestically funded initiatives to expand access to affordable, quality healthcare. Hence, it is important to examine leverage points in aligning the important funding provided by GHIs with UHC oriented health systems to link sustainably with national planning processes.

In this paper, we identify challenges and leverage points for aligning the important GHI funding towards UHC focused health systems in Pakistan, from the perspective of country stakeholders. The paper draws on the Pakistan case study embedded within the global study on the political economy of GHIs [15] commissioned by the consortium on the Future of Global Health Initiatives (FGHI) to help inform the shifts in the long‐term role of GHIs [15] We have three objectives: (1) to understand the contextual ecosystem of GHI assistance to Pakistan; (2) to examine discourses and debates on strengths and weaknesses of existing GHI support in aligning with UHC‐centred delivery; (3) to explore stakeholders interests, power and position in long‐term changes to GHI country assistance. Findings are intended to provide a country‐led perspective on alignment, deepen the understanding of national eco‐systems and a central focus on unpacking of power and pathways to align with longer term primary care priorities.

## 2 Methods

### 2.1 Study Design

A qualitative exploratory country case study approach was adopted to unpack challenges and opportunities of GHIs' assistance for UHC‐centred health systems, drawing on country stakeholders' perspectives and background documentation relevant to the GHI country ecosystem. From the list of six GHIs included in the global study, comprising of GFATM, Gavi, GFF, Unitaid, Coalition for Epidemic Preparedness (CEPI) and FIND, the Global Alliance for Diagnostics ‐ we focused on those with the most substantial country investment ‐ GFATM, Gavi, and the GFF.

### 2.2 Framework

**FIGURE 1:**
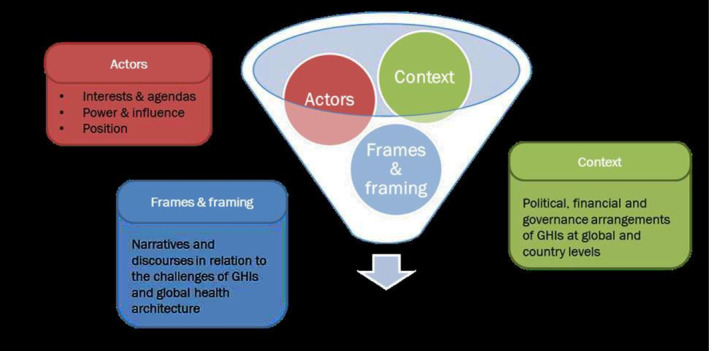
Conceptual Framework: Future of Global Health Initiatives. *Source:* Adapted from Bertone MP, Wurie H, Samai M, Witter S 2018. The bumpy trajectory of performance‐based financing for healthcare in Sierra Leone: agency, structure and frames shaping the policy process. Global Health [Internet]. 2018 Oct 20; 14(1):99.

The examination was guided by a political economy approach (PEA), which is particularly useful to understand policy making by looking at the dynamic process of formal and informal interaction between institutions and stakeholders within given contexts [16]. A PEA framework by Bertone et al. 2018 based on examination of the context, framing and stakeholders for GHI assistance (Figure 1) was applied for systematic probing of the three study objectives guiding data collection and synthesis.

Under this framework the context was interpreted as the country eco‐system within which GHI assistance is provided, comprising of the political decision‐making structure, financial and national planning in Pakistan [17]. Framing was construed as prevalent discourses and debates within the country related to GHI assistance and tensions as well as synergies with UHC progression [17, 18]. Stakeholders were examined in terms of their power, interests, positions for perpetuating existing GHI assistance modalities or bringing in change to progress towards UHC [18, 19]. Power was interpreted as the ability to influence, including both decision‐making powers tied to position of authority as well as soft power related to ability to influence others through coercion, inducement, resources, thought leadership and access to those in power [20].

The PEA elements of context, narratives and stakeholders from Bertone's framework are mapped against each of the three study objectives in Table 1. Research questions, sub‐questions and probes were listed to examine each of the three study objectives drawing on the PEA approach to eliciting tensions, synergies and changes required for progression towards UHC centric health systems (Table 1).

**TABLE 1:** Methods: Research objectives, questions, PEA areas, data sources.

Objectives	Research questions	PEA Areas	Data Sources
1) To understand the eco‐system of GHI funding in Pakistan	1) What are the existing mechanisms for defining and coordinating GHI assistance within the country and key underlying contextual factors?	Underlying country context: Financing structure and modalities; political decision‐making architecture; national health planning; GHIs roles; country stakeholders	Desk review: Online data on health financing/burden of disease; national policies/reform strategies; website content of GHIs and the federal health ministry; independent country assessments/reviews of GHI assistance Key informant interviews: Government planners/leaders: 2 federal, 3 provincial: Total 5 Government disease control managers: 3 federal, 2 provincial: Total 5 Development partners: Total 5 Technical assistance agency: 1 CSOs: 2 Experts: 1
2) To examine discourses an debates on strengths and weaknesses of existing GHI support in aligning with UHC‐centred delivery	2) What strengths and issues are seen linked to GHI ecosystems? 3) What have been the past attempts at change and how these have fared?	Context, framing and stakeholders: Effectiveness and efficiency of funding Alignment with country PHC initiatives/other partners Approach to co‐financing and sustainability Aid accountability: Setting/monitoring of aid targets and disbursements Past efforts to strengthen GHI alignment Lessons from COVID19 pandemic	
3) To explore stakeholders' power, position, interests in long‐term changes required to GHI country assistance	4) Where does power lie to bring about change towards UHC centric systems?	Stakeholders: Explicit and implicit power to bring about change for better alignment Positionality for change: Radical change, little change, no change Underlying interests for change, key enablers to support change	
	5) What longer term changes are required in the GHI ecosystem for progression towards UHC? How can changes be brought about?		

### 2.3 Data Sources

Study participants were domestic policy stakeholders purposely selected based on their experience of working with GHIs and holding a position of seniority within relevant constituencies to provide multiple perspectives. Constituencies included government planning, government disease control programmes, development partners, Civil society organisations (CSOs) inclusive of non‐government organizations (NGOs), technical assistance agencies, and local experts. A total of nineteen country‐based stakeholders were interviewed (Table 1) and effort was made to include both federal and provincial government stakeholders within Pakistan's decentralised health context. Snowballing was applied to recruit new key informant interviews based on suggestions from people interviewed. An open‐ended topic guide was used, organised around the research questions and populated with further sub‐questions and PEA‐guided probes (Table 1). Desk review was undertaken to supplement interview data included: online data from country and global repositories on health financing and burden of disease, national policies and reform strategies, website content of GHIs and the federal health ministry on programmatic initiatives, and GHI assistance reviews solicited from key informants where available.

### 2.4 Data Collection and Analysis

Policy stakeholder views can be difficult to elicit, which can affect the quality of the analysis [21], hence interviews were conducted by three researchers from the country team who were familiar with and had strong links to the local health system. Interviews were separately conducted with common training on the interview tool, interview tool adaptation with mutual feedback after the first interviews and regular meetings to discuss probes and findings were used to ensure consistency of approach across the interviewers. Interviews were conducted with ethics approval from the Aga Khan University Pakistan and the University of Geneva between April to June 2023. Interviews were conducted in‐person as well as online, supported by informed written consent from interviewees, ensuring of privacy and choice of audio recording versus written notes provided to interviewees. Interviews were conducted in‐person as well as online, supported by informed written consent from interviewees. Interviews ranged from 45 to 90 min. Personal identifiers were removed to anonymise the data and to protect the identities of the respondents. Transcript data was deductively coded into main themes corresponding to the research questions and followed by further coding by the three PEA areas of context, framing and stakeholders' power, interest and position.

A triangulation of desk review and interview data was made to synthesise the findings. The country GHI assistance context primarily drew on desk review information supplemented by interviews, whereas challenges, synergies and future vision largely drew on interview data supplemented by documents where available. Synthesis was organised by the three study objectives with highlighting of PEA aspects of stakeholders' interest, power, narratives and context. The researcher consortium met frequently to discuss the emerging findings. Presentation of draft findings was made at two global consultative meetings, and the draft report was circulated to the GHIs prior to finalisation.

## 3 Findings

The findings are categorised by the three research objectives drawing on the PEA framework's area of contextual arrangements, framing discourse and debates and stakeholders' interests, power, positionality for change.

### 3.1 Country Contextual Ecosystem for GHI Assistance

#### 3.1.1 GHI Roles and Funding Modalities

Pakistan is a low middle‐income country with a GDP per capita that has risen from $344 in $1990 to $1365 in 2022 [22]. Communicable and MNCH together comprise of a significant 41% of the health burden with the remaining 59% related to non‐communicable diseases that have rapidly risen over the years [23]. Pakistan remains one of the largest recipients of GHI funding, however national domestic allocations for health in general and preventive health services in particular have remained low (See Box 1) [24]. The country government contributes 57% of funding to the national immunisation programme with a substantial 43% contributed by Gavi [25]. Government contribution for TB, Malaria, HIV is much lower at 20% with a critical 80% borne by the GFATM, and the share has remained static over the years [14]. The government draws on both development and recurrent funding to contribute cofinancing. Development funding is connected to a time bound project, hence providing a projectized approach to programmatic funding, vulnerable to budgetary cuts and delayed releases. In contrast recurrent funding is committed long‐term funding for health care delivery.

**Box 1 Country overview.**

1 Per capita national health spending is US $45 per capita against WHO's recommended US $86 per capita (GoP 2022).

GFATM is the second largest contributor of official development assistance (ODA) to Pakistan accounting for 16% of ODA†

GFATM has disbursed US$ 1.09 billion over 2003–23‡

GFATM support is provided through the platform of the Country Coordination Mechanism based at the federal ministry of health.

CSO *p*Rs and SRs are the major recipients of GFATM country grants.

Federal health ministry is a PR for malaria and TB for an oversight role, UNDP has replaced the ministry as public sector PR for HIV.

Provincial health department are SRs of the federal ministry under GFATM grants.

There is no national policy/join programming document to guide TB, malaria, HIV efforts.

Gavi is the fifth largest contributor accounting for 6.5% of ODA†

Gavi has disbursed US$1.3 billion in assistance from 2001–2019§

National Interagency Coordination Committee (NICC) is the country decision making body for adjustments to GAVI support.

National Immunisation Technical Advisory Group (NITAG) is mandated to provide technical recommendations.

A Comprehensive National Multi‐Year Plan guides national immunisation efforts.

Global Financing Facility has committed $82million over 2023–2027.

Assistance directed through pooled funding as part of World Bank funded National Health Support Programme

Other GHIs: Small‐scale assistance is provided by CEPI, UNITAD, FIND directly to research institutions and NGOs for COVID‐19 and Hepatitis C.

*Sources:* †GoP 2024. Strategic Review of Global Health Initiatives Supported Interventions & Programmes in Pakistan. Evidence for Health Programme, UKAID and Government of Pakistan, December 2024 Islamabad. ‡http://data.theglobalfund.org/location/PAK/financial‐insights; Gavi Annual Progress Report 2023 §https://www.gavi.org/sites/default/files/programmes‐impact/our‐impact/apr/Gavi‐Progress‐Report‐2023.pdf.

Gavi has disbursed US$1.3 billion in assistance from 2001–2019, with additional funding for COVID‐19 during the pandemic years [25]. The financing model comprises off‐budget support grants to UN agencies, international suppliers and CSOs as well as co‐financing of vaccines with country governments. The major share of assistance to Pakistan (80%) is spent on financing vaccines (Table 2). Basic vaccines are now fully financed by the country government (Box 1). Gavi assistance faces issues of sustainability and weak government oversight on the delivery of immunisation services. Gavi support in absolute amount has grown over the years despite declining of share of funding from 98% in 2016 to 43% in 2023 due to addition of new Gavi supported vaccines in the routine immunisation schedule. Introduction of some vaccines without local cost effectiveness data has been contended by country finance and planning departments raising concerns of widening liabilities for vaccine financing. Health Systems Strengthening (HSS) and immunisation support receive a smaller share of Gavi funding (Table 2) but are instrumental in prioritising under‐served areas. However, the routing of HSS and immunisation services support is through UN agencies, limiting control and ownership of the expanded programme of immunisation.

**TABLE 2:** National health burden and country assistance by GHIs.

Burden of disease	Funding support
Fully immunised children: 77%	GAVI: $2487million† (2000–2027 commitment) Funding distribution by proportionate share and country partners: Vaccines (80%)—UNICEF: 1) BCG, OPV, DPT vaccines ‐ fully financed by government 2) PCV, IPV, Penta, Rota, Typhoid, HPV vaccines ‐ main financing by GAVI, partial co‐financing by country government HSS (10%): Additional vaccinators, community mobilisation/campaigns, reporting, MIS: WHO and UNICEF Immunisation services support (4%): Programmatic staff, technical assistance, training ‐ WHO‐UNICEF; INGOs Cold chain (0.4%): Equipment/warehousing: WHO Civil society support (0.4%): Additional vaccinators, health education/awareness, data analytics ‐ INGOs, local CSOs Vaccinators, cold chain, health infrastructure, supervision, training—Government
Tuberculosis (TB) cases in the country: 264 cases per 100,000	GFATM: $148 million^a^ (2021–2023) Funding distribution by proportionate share and country partners: Procurement of TB diagnostic and treatment commodities, diagnostic equipment including GeneXpert machines for dedicated TB sites– Government PR Transportation, warehousing support—CSO PR (INGO), government PR Private provider integration for TB‐DOTS, mobile vans for case finding, case reporting, health education/awareness, supervision, training—CSO PR (INGO)
Malaria cases in the country: 427,000	GFATM: $34 million^a^ (2021–2023) Funding distribution by proportionate share and country partners: Antimalarial drugs and diagnostics, surveillance, case reporting, training of outreach workers—Government PR Transportation, warehousing support—Government PR, CSO PR (local NGO) Bed nets distribution, case reporting, health education/awareness, training to private providers, programmatic support—CSO PR (local NGO)
HIV/AIDS cases in the country: 210,000	GFATM: $69.5 million^a^ (2021–2023) Funding distribution by proportionate share and country partners: Harm reduction for injectable drug users, warehousing support—CSO PR (local NGO) Harm reduction for other high‐risk groups, opioid therapy, health awareness, self, testing kits, warehousing support ‐ UNDP Commodities and logistics for ART centres—Government PR
MNCH Maternal mortality Rate (MMR): 154 per 100,000 Infant mortality Rate (IMR) per 100,000	Global financing Facility: $82million§ (2023–2027) Funding emphasis and modalities: Funding to support institutional deliveries, quality care, affordable access in deprived areas through public health sector infrastructure Funding linked to process and output‐based deliverables Funding support for MNCH design, monitoring and evaluation

*Source:* †GAVI. https://www.gavi.org/sites/default/files/programmes‐impact/our‐impact/apr/Gavi‐Progress‐Report‐2023.pdf.

^a^ GFATM https://data.theglobalfund.org/grants. GFF. https://data.gffportal.org/country/pakistan.

GFATM has disbursed US$1.1 billion since 2003–2023 with the heaviest investment in TB ($0.7 billion), followed by malaria ($0.2 billion) and HIV/AIDS ($0.16 billion) [26] (Box 1). GFATM assistance faces a considerable challenge of sustainability with its absolute funding growing over the years, however government contributions remaining static at 20%. The country is majorly reliant on GFATM for commodities, outreach activities and programmatic staff (Table 2). The grant‐based funding model has faced issues of fragmented responsibility and contention on roles. Country grants are provided to CSOs as principal recipients (PRs), whereas programmatic support for extra staffing and equipment flows through a public sector PR under the oversight of the Country Coordinating Mechanism (CCM). There has been contention between the government and the GFATM on the selection of CSO principal recipients (PRs) and the suspension of CCM's mandate of identifying and overseeing *p*Rs by GFATM since 2020 as a result of poor country portfolio performance [14]. The ministry is the public sector PR for TB and malaria but has been replaced by the UNDP as public sector PR for HIV/AIDS. Additionally, CSO *p*Rs for each of the three disease portfolios undertake considerable service delivery.

GFF assistance was initiated in 2023 with commitment of $82 million over four years for MNCH as part of a World Bank led pooled fund for primary care (Box 1). MNCH commodities, staff and equipment have been traditionally financed by the country government without donor assistance but there are resource gaps in underserved areas (Table 2). This will be disbursed as part of a larger World Bank managed pooled funding initiative to support recurrent budgets of provinces for essential primary health care services [27].

#### 3.1.2 Political Decision‐Making Architecture

Disease control stewardship in decentralised Pakistan necessitates bidirectional engagement with both federal and provincial governments. The devolution of 2011 was an important constitutional milestone, discontinuing mandate and funding for 11 vertical disease control programs based at the federal health ministry. It shifted the federal role to national oversight and made provinces responsible for resourcing, planning and delivery [28]. Hence the budget of the federal health ministry also got significantly reduced with increase in fiscal space of the provinces [29]. Provinces particularly have more recurrent funding available as part of the primary care and hospital care delivery system that falls under provincial mandate and hence better fiscal space to absorb disease programming requirements.

GFATM and Gavi were slow and reluctant to engage with provinces post‐devolution in contrast to other development partners shifting to support PHC strengthening in the provinces [30]. Gavi has slowly adapted to include provinces in aid programming discussions, necessitated also by provincial governments being co‐financers of Gavi supported vaccines. However, routing of GAVI HSS support through UN agencies, rather than provincial and federal governments, has been a bone of contention. GFATM driven by stringent grant accountability requirements helped re‐establish and centralise TB, Malaria, HIV programmes under the Common Management Unit (CMU) within the federal health ministry in 2018. Provincial governments despite having better resources and a more powerful mandate for service delivery serve as Sub‐Recipients (SRs) under the federal ministry with low decision space over GFATM supported programming. The verticalized projectized fund flow also does not create incentives for provinces to integrate within recurrent PHC budgets.

GFF's approach is a departure from the Gavi and GFATM provides direct resourcing to provincial health departments as part of the multi‐donor pool to support planning and delivery of essential primary care services.

Gavi has a relatively better approach, the government is involved, and budget has changed from development [projectised] to the recurrent side. NHSP (GFF) support is through recurrent budget and government will invest and sustain the investment. GFATM is still facing the problems of effectiveness and sustainability.

(Government planner)

#### 3.1.3 National Health Planning

Gavi and GFATM have well defined aid approval processes in Pakistan to support selected activities necessary for portfolio delivery (Box 1), but these have operated separately from national policy and UHC reforms process. Pakistan has an overarching national health vision emphasising primary healthcare [31] and provincial health sector strategies based on integrated primary care within provincial reforms. There were attempts to translate health sector strategies into long‐term financial planning but were disrupted due to continuation of federally led GHI support, competing initiatives by different development partners as well as changing health priorities of successive political governments. In recent years there has been a domestically led UHC momentum involving a national health insurance initiative, establishment of healthcare regulatory authorities, public private partnership initiatives for strengthening primary‐secondary care and development of an essential health service package. The UHC reforms are designed and funded by the provincial and federal governments, but face resource gaps in scaling up.

Long terms planning and budgeting for 10–15 years is not envisioned most of the time, although health policies and strategies are developed and available at national and provincial level. Compliance and implementation of health sector strategies and policies has been the greatest challenge over the past years.

(CSO)

Although GHIs, UN agencies, development banks and bilateral aid agencies have historically competed for influence with the government there has been growing alignment since 2021 to coordinate influence with the government. Eight partner agencies, including GFATM, Gavi and GFF, have established a dialogue forum for SDG3 Global Action Plan for Healthy Lives and Well‐being for All (GAP) led by the WHO to create synergies on aid coordination. Dialogue is slow, engagement is mainly confined to the federal ministry not extended to the provinces and is dependent on committed staff members within partner agencies, rather than institutional incentives to collaborate [32]. As a test case, both Gavi and GFATM have agreed to direct $20 million and $5 million respectively through the World Bank‐GFF supported pooled funding platform called the National Health Support Programme (NHSP) launched in 2023, while continuing to provide the major share of funding through parallel country support.

### 3.2 Discourse and Debates on Strengths and Challenges of GHI Support in Pakistan

The existing framing on GHIs, in terms of policy discourse and debates, was unpacked to highlight key strengths and challenges as perceived by country stakeholders.

#### 3.2.1 Programmatic Efficiency and Impact

Country stakeholders credit GHIs for increasing policy attention to communicable diseases and making gains in disease coverage. GHIs' technical expertise for communicable diseases metrics and planning, as well as the provision of visibility to marginalised populations, is well recognised. Disease managers particularly stress the critical financing of quality commodities and processes to ensure transparency of spend, particularly in the case of GFATM (Table 3). However, almost all stakeholders considered that gains in disease coverage were insufficient compared to how much aid has been spent. For example, public sector health planners pointed to gains in skilled birth delivery coverage that have occurred with horizontal delivery, questioning the value for money of verticalized GHI aid assistance. Yet others pointed to the resource gaps for countering steep rise in NCDs through primary care services, emphasising that the verticalized implementation approach based on siloed single disease models has outlived its utility.

For Infectious diseases, the biggest funding is coming through the Global Fund [GFATM, Gavi] without which health indicators could have gone further down. But there is poor performance for infectious diseases. RMNCH is implemented through the integrated mechanism, maternal mortality has declined and skilled birth attendance has increased.

(Government planner)

**TABLE 3:** Discourse and debates on strengths and weaknesses of GHI support.

Context	Strengths	Weaknesses
Low government funding to preventive health Weak PHC stewardship, frequent leadership changes Weak long‐term financial planning for health Federal‐provincial government power tussles over legitimacy and resourcing Multiple GHI and development partner funding streams create coordination challenges Large role of UN agencies and CSOs with GHI assistance Competition for influence by development partners, transitioning to search for synergies for PHC under UHC	GHI prioritisation of over‐looked communicable disease control/preventive health	Questions on value for money Lack of links with disease with similar routes of spread Cross‐programmatic inefficiencies due to duplicative resourcing GFATM systems more centralised, de‐linked and slow moving, better adaption of GAVI
	GHI assistance coordination initiated with other development partners for PHC under UHC	Parallel GHI funding to INGOs, UN partners & CSOs disincentivises links with country PHC‐UHC planning/budgeting Lack of local capability for tracking aid data and financing metrics disempowers government
	Consistently high spending by GHIs	GHIs assistance used as ad hoc funding by country planners, resulting in weak growth of domestic funding for communicable disease control
	Technical leadership for disease metrics, tools, monitoring	Globally driven solutions rather than locally derived Emphasis on disease burden metrics and projectized solutions, fragmented systems support
	Expansion from traditionally centralised dialogue to increasing dialogue with sub‐national governments	Lack of links with recurrent budgets financed by provincial governments Weak links with planners and reform initiatives Federalised CCM GFATM model compromises provincial autonomy

Efficiency concerns for Gavi and GFATM assistance were largely related to cross‐programmatic inefficiencies. Stakeholders pointed to the uncoordinated proliferation of community health workers and vaccinators, multiple community engagement initiatives and parallel information systems. Development partners, government planners and CSOs called for widening the reach to multiple diseases by reducing parallel GHI supported district supervisory systems and consolidating HR resources. The proliferation in technical committees for priority health issues supported by GHIs, development partners and UN agencies, also made it extremely challenging for all country stakeholders to navigate information and planning for synergised disease control.

We have just done some mapping, and we realize there are 17 different forums at the federal level only and maybe the provincial level as well. We do not know how many coordination mechanisms exist. Unfortunately, we don't have that (single country plan) for Pakistan. So, we have for GFATM separate, for NHSP separate one and for Gavi separate one and then maybe for polio a separate one. Therefore, even information is not being shared.

(development partner)

There were also operational efficiency issues reported mainly with the GFATM assistance model. These included time‐intensive funding applications, multiple grant cycles and heavy accounting requirements. Commodities being restricted to grant‐supported districts, rather than provided across the country as in the case with Gavi supported vaccines also created budget alignment challenges. Diversification of some GFATM funding into the World Bank‐led pooled support to recurrent financing was meant to be a test case for a more timely and agile flow of funding for wider essential healthcare services.

#### 3.2.2 Alignment of GHIs With National Priorities

Country stakeholders acknowledged an improvement in coordination between GHIs and development partners but alignment with national policy processes was perceived to be weak. Targets, approaches and solutions supported by GHIs were perceived to be driven by global priorities driven by global researchers and disease outcomes rather than health locally innovative solutions based on country deliberations supported by process‐based targets. Stakeholders also pointed to weak alignment with other endemic infectious diseases that had common pathways of spread. For example, Pakistan faces frequent serious outbreaks of dengue, but GHI funding remains confined to malaria. Similarly, there was endemicity of Hepatitis B and C infections spread by unsafe needle use [33], but harm reduction assistance to counter unsafe needle use was only targeted towards the smaller number of HIV cases. Furthermore, GHIs assistance was not perceived to be meaningfully linked to local UHC reforms such as implementation of the essential health care package, public‐private partnership contracts for primary care and regulation of health services, resulting in missed opportunities for political support and sustainable financing. For example, a benefits package for an essential healthcare had been developed under government coordination but faced budgetary gaps for roll‐out [34]. One of the reasons for this disconnect was the primary engagement of GHIs' with disease managers, with more perfunctory dialogue held with PHC planners, however disease managers did not have the mandate for PHC reforms and were often blamed for pursuing their own agenda of securing disease specific funding.

On paper, dialogue is carried out with the bureaucracy [secretary]; but practically the dialogue is done with the programs which do not consider the other aspects e.g. challenges, sustainability and duplication dimensions.

(Government planner)

When they [CCM] are putting in [GFATM] applications, their objective is not reform, their objective is only to secure funding.

(CSO)

Ability to provide flexible financing in response to new, urgent country needs was another area raised by country stakeholders, considering recent disease outbreaks. GAVI's agility in mobilising vaccines for the COVID‐19 pandemic was noted as a key strength and attributed to its co‐financing funding stream (Table 3), whereas the GFATM entirely dependent on grant‐based model had been slow‐moving in responding to new pandemic raised needs that were not built into pre‐programed cycles.

#### 3.2.3 Approach to Co‐Financing and Sustainability

Sustainability of GHI assistance was brought up by all categories of stakeholders. There was realisation that GHI assistance is insufficient to meet the huge country population and future aid reduction is likely with Pakistan's transition to a middle‐income country. There was concern that multiple fragmented funding initiatives have contributed to the government perception of GHIs assistance as an ad hoc opportunity for disease control. Country stakeholders blamed failure of long‐term financial planning by the government for primary healthcare and poor preparation for transitioning away from external assistance. The parallel funding to INGOs, CSOs, and UN agencies led to compartmentalisation of this funding, de‐linking from country financial planning for PHC under UHC. Furthermore, the grant cycles of GHIs did not coincide with annual budgetary planning in Pakistan, making it difficult to align spending and hence perpetuating a compartmentalised approach.

This money [through GHIs] is limited and not forever; it will exhaust one day because it is an interim funding. We have to improve internal funding to sustain the interventions when funding will be withdrawn. We do not see it from this angle and have not done planning with this angle. We need to see that whatever money is coming through funding, how can we use this opportunity to improve the domestic financing.

(Government planner)

#### 3.2.4 Accountability for Aid Disbursements

There were vociferous complaints by government planners and disease managers about the lack of data on GHI support within the government. Stakeholders pointed to reluctance of GHIs to share data on parallel funding and available records of direct support to the government were fragmented across various government entities. This made it impossible to ensure accountability by GHIs even for motivated leaders. Technical assistance provided by GHIs was provided through short term support for human resource and service delivery, with very little capability built on disease expenditure and budgeting analysis. Public finance management (PFM) capacity was strikingly absent across both disease programs and PHC planning units. An earlier attempt to provide performance‐based immunisation financing by the World Bank had faced challenges due to poor public finance management capacity to analyse and report expenditure for deliverable based financing [35]. With considerable GHI assistance flowing through UN agencies, there was often confusion on which is the actual entity to hold accountable. Frequent leadership transfers within the governments further eroded institutional capacity, making it almost impossible to establish a sustained cohesive dialogue between government and partners on setting aid targets and accountability:

Transfers in the bureaucracy are just crazy because then you have to start again. We should just record this (performance‐based financing) on tape and then press play every time we have a new secretary, a new DG.

(Development partner)

### 3.3 Stakeholder Power, Position and Interests for Long‐Term Changes to GHI Country Assistance

Perspectives of federal and provincial stakeholders on long term changes to GHI country assistance was explored, unpacking ability to exert power and authority for changes, position in terms of continued GHI role and underlying interests.

#### 3.3.1 Power to Bring About Changes

Most stakeholders felt that authority to articulate priorities lies with the government but is weakly exercised (Table 4). Low political interest in disease control and entrenched long‐standing reliance on the notion of ‘free money’ from GHIs had together contributed to weak exercise of stewardship. Lack of consolidation of aid data further weakened the power of the government to question and align funding assistance. GHIs were also blamed for not being forthcoming on sharing data on allocation and expenditures. Power had hence gravitated towards the GHIs and was directly exercised by GHIs through control of substantial funding and bringing the country to account through performance reviews. It was also exercised through technical leadership provided through consultants to support the CCM and UN agencies to support the immunisation programme. In contrast, power within government stakeholders had drifted towards government disease managers. Officials in charge of PHC planning had high power, albeit weakly exercised due to low interest, weak understanding of priority health issues and lack of aid data to exert accountability. Disease programme managers had lesser mandate to align GHIs but exerted power as the main holders of GHI country dialogue. Development partners exercised power linked to PHC resourcing and coordination but had limited influence as development aid was only marginal compared to domestic financing of the health sector. CSOs had low power, being directly dependent to GHIs for funding and accountability, and not systematically engaged in government planning.

The power lies with GHIs so far. They send you the support but we don’t have a say. If you don’t have a say, you don’t have power. We appreciate the support because it is filling our gaps. For better results we need coordination.

(Government disease manager)

Power lies with the body who has the money; Global Fund, Gavi. We have to adopt the rules of GHIs. Coordination is with the government but information is lacking. The accountability …donors do not proactively share their information on allocation and expenditure. Data is not available.

(government planner)

**TABLE 4:** Stakeholders' Power, Positionality and Interests for Future UHC aligned GHI Assistance.

Stakeholders	Power to change GHI ecosystem	Positionality: Preferred future ecosystem	Interest in changing GHI ecosystem	Enablers for change
GHIs	High power linked to speciality funding, global leadership on disease narratives, country performance reviews	Verticalized and selective support, growing openness to diversifying funding	Visibility, global disease stewardship, headquarter‐based funding models	Link to government led UHC momentum for country ownership Shift accountability to both GHIs and country for better delivery Growing data analytics capability for aid stewardship Introducing collaborative disease governance model with incentives across federal and provincial levels
Government disease managers	Medium power linked to brokerage of country disease control dialogue, technical voice guiding PHC planners	Continue GHIs' support but build local accountability, locally derived solutions/targets, better efficiency	Visibility of disease control and programs, greater government voice in aid coordination, diversify assistance to include provinces	
Government planners	High power linked to country priorities and accountability mandate but weakly exercised	Horizontal disease integration with planning and analytics support	Alignment and efficiency across multiple competing PHC needs, bridge financing for PHC, diversify assistance to include provinces	
CSOs, experts, technical assistance agencies	Low power, dependency on GHI funding, lack of systematic involvement in country dialogue	Programmatic synergies across multiple funding streams	Maximise CSOs across multiple diseases for underserved communities	
Development partners	Medium power linked to PHC coordination and limited resourcing support to country government	Horizontal coordination for PHC, focus on health systems processes for disease control, grow domestic financing	Amplify influence on government through joint resourcing	

#### 3.3.2 Positionality to Bring About Changes to GHI Country Assistance

Stakeholders commonly emphasised that Pakistan would need GHIs' support in the long term but with a fundamental shift in the modus operandi of GHIs. Disease managers were positioned to support a longer‐term vision based on greater voice, coordination and accountability on disease programme funding by disease managers (Table 4). Other stakeholders, including PHC planners, development partners and CSOs, viewed future success as involving a shift from vertical to integrated PHC delivery, impactful planning coordination, investment in accountability frameworks and bridge financing to meet targets (Table 4). Differences were also seen between provincial and national stakeholders, with provincial stakeholder positioned towards greater local voice within future arrangements. Stakeholders considered links to UHC, led and financed by the country government, as critical to secure sustainable country ownership and financing. Although national UHC efforts were confined to affordable hospital care, there was growing policy maker interest in operational models for affordable primary care.

The need for GHIs will remain there in future. Even if the funding is not needed; technical assistance, expertise and technology will be needed from the GHIs.

(development partner)

Change vertical approach to horizontal integration, we need analysis, we need a coordination mechanism. When reforms come, we need finance [to implement reforms]. All reforms must cut across from both sides i.e. recipient and donors.

(Government planner)

There were differences of opinion on whether all GHI or only partial GHI assistance should flow through public sector budgets. Certain government stakeholders held more assertive views that government should be the only direct recipient, with CSO disbursements to be managed by the country government. Yet others were inclined towards GHI or third‐party disbursement to CSOs but within a converged planning framework. Government stakeholders emphasised building understanding within GHIs of the public sector financing process to reduce reluctance to engage, align and contribute to government budgetary cycles.

First reform is that now the funds/money from funding agency are being sent directly through the government channel for the first time. The second reform should be that the GHIs understand the procedures and processes of the government… GHIs people are technically sound but are not familiar with government procedures.

*(Government disease manager)*

#### 3.3.3 Stakeholders' Interests for Change

The positionality of stakeholders was influenced by underlying interests for change (Table 4). Dwindling of aid resources was a common driver to move towards stronger integration and co‐financing. Government planners were interested in meeting multiple PHC needs, with GHIs' funding to support fiscal gaps in integrated programmes. Disease control managers were interested in exerting local accountability on GHI assistance and ensuring alignment within local programmatic planning. Within the government, federal government interest was in remaining relevant and provincial governments interest in exertion of legitimacy. CSOs were interested in maximising reach across diseases for underserved communities, supported by over‐lapping projects, but acknowledged low political voice within the reform process. Development partners wanted amplified influence through joint resourcing of PHC and for budget support to be linked to performance accountability for delivery.

Within the bigger construct, we see more money going to health through domestic resources, because in a country like Pakistan, donor resources could never be enough to satisfy the [country’s] needs.

(Development partner)

#### 3.3.4 Enablers for Change

Stakeholders proposed enablers for meaningful future alignment and integration of GHI assistance. Linking to the government led UHC agenda was considered important to gain political ownership and link with domestic financing. Investing in aid data and metrics to ensure accountability and alignment of GHI assistance was also raised by different stakeholders, with emphasis on building capability in analytical tracking. Stakeholders pointed to government ownership, federal‐provincial coordination and use of digital data that helped the country government exert transformational leadership during the COVID‐19 pandemic. There was emphasis on creating a collaborative governance paradigm for both federal and provincial government to exert respective roles, overcome territorial tensions and allow direct flow of funds to the provinces.

Actually, it is a game of jurisdiction and powers. We don’t like to give up our powers. If something is in our ambit of work, we don’t want any other to do that task. There should be a collaborative paradigm, collective wisdom and common goal. We have territorial issues in doing work and services.

(Government planner)

Finally, sharing joint responsibility for targets by the GHIs and the government was proposed as another area that may support future alignment and better horizontal integration. At the same time there were country concerns on GHI's interest in visibility, and scepticism of GHIs buy‐in for sustainable UHC integration. Aid incentives were pointed out to be perversely geared not to reward countries for sustainable transition, but perpetuating dependency on GHIs and other external funders.

GHIs have interest to remain visible and don’t want to disappear…..Pakistan is not that much empowered in narrating priorities compared to other regional countries…. Globally incentives are few for the recipient countries (that achieve aid targets). What is the incentives for the Sri Lanka which has maternal mortality as good as Scandinavian countries? There are conflicting priorities in funding agencies.

(CSO)

## 4 Discussion

The paper emphasised an urgent re‐thinking of GHI assistance in Pakistan for accountability to domestic stakeholders, alignment with country's fiscal planning for PHC under UHC and building financial sustainability. A political economy framework helped in unpacking contextual tension points within GHI assistance, existing country discourse on strengths and weakness, stakeholders' power, interest to enact changes and positionality for future changes to the role of GHIs. Lessons are salient for other LMICs dependent on GHIs, given the recent rapid disruptions in global aid.

### 4.1 Contextual Tensions

The paper highlighted an expanding GHI mandate despite Pakistan's steady growth towards a middle‐income country status. Contextual weaknesses of headquarter‐led planning, inefficient disease specific funding, and parallel off‐budget projectized support by GFATM and Gavi, combined with country challenges of political complacency on aid reliance for disease control and federal‐provincial competing interests. Resulted in weak alignment of cohesive direction for GHI assistance within UHC. Expansion in mandate of GHIs accompanied by proliferation of UN partners, international suppliers and large NGOs, further clouded authority, roles and jurisdiction, cyclically reducing government capability to exert aid accountability. Attempts at PHC stewardship had been disrupted by verticalized engagement of GHIs and absence of aid data tracking systems. Technical assistance from GHIs was not geared to prepare for alignment and transition, limited to short‐term service delivery support overlooking longer‐term gains from investing in financial planning capability.

#### 4.1.1 Existing Discourse and Power‐Imbalances

Country stakeholders preferred budgetary co‐financing as a pathway to exert local accountability, fiscal efficiency and cohesive approach to disease financing. Concerns were expressed with all GHI models. GFATM assistance, dependent entirely on the grant‐based model faced the most concerns of alignment with integrated PHC financing and weak agility to adapt to shocks. GAVI's vaccine co‐financing approach resulting in successful full financing of basic vaccines by Pakistan was more successful in building ownership and self‐reliance than parallel off‐budget support to suppliers that centralised power with Gavi headquarters disempowering domestic decision making. GFF on‐budget financing to catalyse recurrent budgets was an innovative recent departure but its reliance on deliverable linked indicators (DLIs) without building PFM capacity support constrained chances of decisive leadership from domestic stakeholders [36].

#### 4.1.2 Stakeholders' Power, Interest and Position for Future Changes

Declining aid funding served as a common interest across all categories of stakeholders for a counter‐narrative on future role of GHIs. Long‐term vision of change involved continued role of GHIs with funding reconfigured to support domestic financing under PHC planning and accountability of GHIs to country stakeholders. Power and interest of stakeholders influenced their positionality on a counter‐narrative for the future. Disease managers wanted local planning to set the aid direction, strong coordination on grants to non‐state actors and on‐budget support to individual disease control budgets for improving synergies but also protecting their own leadership power. Other stakeholders including PHC planners, experts, CSOs, and development partners favoured more radical power changes for cross‐programmatic synergies of on‐budget support to horizontal PHC budgets for better efficiency accompanied by catalytic disease financing targets for the country government for increasing self‐reliance.

Global discourse has raised concerns of fragmentation created with skewed systems support by GHIs [37, 38, 39, 40]. In‐depth country case studies are mainly from Africa and draw on examination of a specific initiative such as the PEPFAR, GFATM or Gavi highlighting parallel GHI funding and verticalized governance structures as the principal cause of inefficiency and weak sustainability in aid‐dependent countries [41, 42, 43, 44, 45, 46]. Our findings provide a timely perspective from domestic stakeholders on appetite and margins for cross‐cutting change across GHIs for LMICs. A counter‐narrative from Pakistan of shared GHI‐country accountability, developing disease‐UHC links, catalytic on‐budget support for financial sustainability and building PFM capability for more efficient aid management is applicable to other similar LMICs and resonates with recent evidence on optimising UHC initiatives [47, 48].

The Lusaka Agenda picks up some of these key areas for a future shift in the role of GHIs, proposing measures for defragmenting funding of PHC systems and metrics for tracking and accountability [49, 50].The recent cuts to aid funding, geopolitical preference for domestic over international priorities for aid spending and a retreat from multilateralism, can have cascading consequences on GHIs and LMICs [51, 52]. For far too long the GHIs dominance of country disease planning and funding has inadvertently dwarfed the development of country resilience and diversification of funding^,^ [53]. Given recent unpredictable changes, an urgent shift in GHIs role and assistance models is required to help build local capability and maximise impact and efficiency of anticipated aid reductions in LMICs.

Limitations: The rapid timeline for the study limited the amount of data collected, however we rapidly achieved saturation of findings suggesting that key themes were not missed in the analysis. The study only included the viewpoints of country‐based stakeholders, not GHIs which can bias the analysis. However, comments of GHI country teams were included in the finalisation of the case study and interviews with GHI staff in Geneva were included in the global study providing a range of self‐critical and supportive positions.

## 5 Conclusion

We conclude that GHI assistance while important in achieving disease gains is not geared to prepare Pakistan and similar LMICs for sustainable, impactful PHC financing. The GHI led grant based parallel funding model erodes government leadership, fragments accountability, and results in inefficient management of scare resources. Addressing national power imbalances and re‐framing national discourse must be at the centre of paradigm shifts in country assistance by GHIs, although contextual modalities will differ across countries. Future reform to GHI assistance include establishing links with UHC stakeholders and initiatives, fewer intermediaries, catalytic support to grow domestic budgets and PFM support for aid management. Urgent actions are required within the current context of changes in global aid to build local capability for easing dependence on GHIs while protecting equitable gains in disease outcomes. The political economy approach is useful to identify country entry points in LMICs for implementation of the Lusaka agenda.

## Author Contributions

SZ led on paper concept and draughting. methodological approach and design were led by SW and KB. data was collated by SZ, SSH, AS, ZS. analysis critically reviewed by SW, VR, NP, RJ, KB. all authors contributed to various draughts of the paper and approved the finalised version.

## Funding

The findings are drawn from a Wellcome Trust grant to the University of Geneva on Reimagining the Future of Global Health Initiatives delivered through a research consortium with Pakistan data collated by the Aga Khan University. The views expressed here are solely of the authors and not of the funders.

## Ethics Statement

Ethical approval was obtained from the Aga Khan University Ethics Review Committee‐Social Sciences, Humanities and Arts (057‐ERC‐SSHA‐2023) and the University of Geneva.

## Conflicts of Interest

Authors confirm that there are no competing interests.

## Data Availability

The data that support the findings of this study are available on request from the corresponding author. The data are not publicly available due to privacy or ethical restrictions.
